# Supplementary figures and images for: Environmental DNA as a complementary tool for biodiversity monitoring: A multi-technique and multi-trophic approach to investigate cetacean distribution and feeding ecology
Source: PLoS One. 2024 Oct 16;19(10):e0300992. doi: 10.1371/journal.pone.0300992 (PMC11482729; doi:10.1371/journal.pone.0300992)

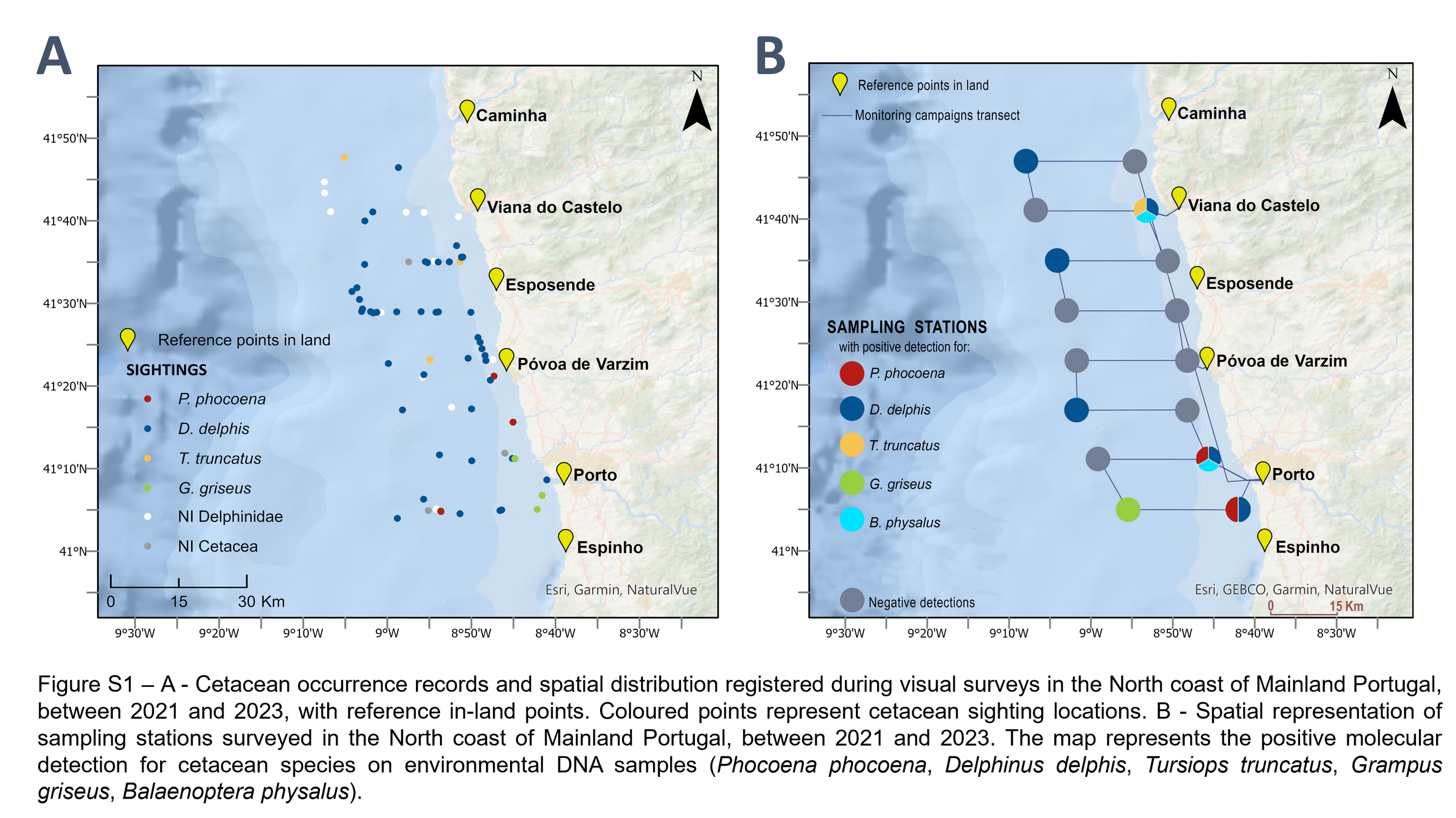

Supplement: S1 Fig — A—Cetacean occurrence records and spatial distribution registered during visual surveys in the North coast of Mainland Portugal, between 2021 and 2023, with reference in-land points. Coloured points represent cetacean sighting locations. B—Spatial representation of sampling stations surveyed in the North coast of Mainland Portugal, between 2021 and 2023. The map represents the positive molecular detection for cetacean species on environmental DNA samples (Phocoena phocoena, Delphinus delphis, Tursiops truncatus, Grampus griseus, Balaenoptera physalus). (TIF) [file pone.0300992.s001.tif]
